# Melatonin, Caffeine, or Their Combination: Effects on Sleep, Performance, Perceived Exertion in a Placebo-Controlled Crossover Study

**DOI:** 10.3390/nu18091425

**Published:** 2026-04-30

**Authors:** Nourhène Mahdi, Slaheddine Delleli, Khouloud Ben Maaoui, Arwa Jebabli, Juan Del Coso, Hamdi Chtourou, Luca Paolo Ardigò, Ibrahim Ouergui

**Affiliations:** 1 High Institute of Sport and Physical Education of Sfax, University of Sfax, Sfax 3000, Tunisia; nourhene648@gmail.com (N.M.); sdelleli2018@gmail.com (S.D.); benmaaouikhouloud88@gmail.com (K.B.M.); jebabliarwa@gmail.com (A.J.); h_chtourou@yahoo.fr (H.C.); 2 Research Laboratory Education, Motricity, Sport and Health, EM2S, LR19JS01, University of Sfax, Sfax 3000, Tunisia; 3 Physical Activity, Sport and Health, Research Unit, UR18JS01, National Sport Observatory, Tunis 1003, Tunisia; 4 Sport Sciences Research Centre, Rey Juan Carlos University, 28943 Fuenlabrada, Spain; juan.delcoso@urjc.es; 5 Department of Teacher Education, NLA University College, 0166 Oslo, Norway; 6 High Institute of Sport and Physical Education of Kef, University of Jendouba, Kef 7100, Tunisia; 7 Research Unit Sport Sciences, Health and Movement, UR22JS01, University of Jendouba, Kef 7100, Tunisia

**Keywords:** ergogenic aid, muscle damage, inflammation, sleep, sports performance

## Abstract

**Background/Objectives**: Melatonin (MEL) promotes sleep and recovery, while caffeine (CAF) enhances alertness and performance. Despite their common use among athletes, their potential interaction remains underexplored. This study examined the effects of MEL and CAF, administered separately or in combination, on sleep, physical performance, physiological, biochemical, and perceptual responses in trained males. **Methods**: In a randomized double-blind placebo-controlled crossover study, fourteen trained males (22.4 ± 2.9 years) underwent four conditions, designed to isolate the effects of each substance and their interaction: (1) PLA + PLA: placebo before sleep and placebo in the morning; (2) PLA + CAF: placebo before sleep and caffeine (3 mg·kg^−1^) in the morning; (3) MEL + PLA: melatonin (6 mg) before sleep and placebo in the morning; and (4) MEL + CAF: melatonin before sleep followed by caffeine in the morning. One hour after the morning ingestion, participants performed the 5 m shuttle run test (5mSRT). Blood samples were collected pre- and post-exercise to assess markers of muscle damage (creatine kinase, lactate dehydrogenase, aspartate aminotransferase, and alanine aminotransferase) and inflammation (C-reactive protein). Peak heart rate (HR_peak_) and rating of perceived exertion (RPE) were recorded throughout the test. Sleep was assessed only during the night following melatonin or placebo ingestion. **Results**: No differences were observed in sleep parameters between conditions (*p* > 0.05). Total distance in the 5mSRT increased following MEL + CAF and PLA + CAF conditions compared with PLA + PLA. Moreover, MEL + CAF reduced muscle damage and inflammation markers compared with PLA + PLA, MEL + PLA, and PLA + CAF conditions (*p* < 0.05). **Conclusions**: The ingestion of nocturnal MEL and next-day CAF was associated with improvements in certain high-intensity exercise performance outcomes, along with changes in muscle damage and inflammation.

## 1. Introduction

Melatonin is a methoxyindole (*N*-acetyl-5-methoxytryptamine; MEL) primarily synthesized from serotonin in the pineal gland [1]. Its production follows the light–dark cycle, resulting in an increased production at night and a decreased production during the day. Light acts as both a masking agent and a primary stimulus for the circadian system, acutely influencing sleep–wake behavior while also entraining endogenous circadian rhythms [2,3]. MEL levels typically peak between 02:00 and 04:00 h, which is essential for maintaining synchronized biological cycles [4]. MEL is a chronobiotic that influences the body in several essential ways for exercise and recovery and is a highly effective antioxidant that protects cells from damage induced by reactive oxygen species (ROS) and reactive nitrogen species (RNS), particularly within mitochondria [5]. MEL has been shown to lower inflammation, hence facilitating muscle recovery following intense exercise [6]. Recent research indicates that nightly MEL supplementation may enhance next-day exercise performance. Administering MEL promptly has also been demonstrated to assist with both short-term and long-term tasks, including aerobic and anaerobic exercises [7]. However, few studies have examined the impact of night MEL intake on exercise conducted in the morning of the next day. Cheikh et al. [8] conducted a study demonstrating that adolescents who ingested 10 mg of MEL the night before performing the running-based anaerobic sprint test (RAST) exhibited a significant reduction in cellular damage and inflammatory biomarkers. In a similar study, participants ingested the same dose of MEL (10 mg) after performing the Yo-Yo intermittent recovery test level 1 (YYIRT-1). Supplementation improved both sleep quantity and quality during the subsequent night and enhanced physical performance the following morning, including vertical and horizontal jumps (VJs, HJs) and handgrip strength (HG), and the five-jump test (5-JT) was improved [7].

Caffeine (1,3,7-trimethylxanthine; CAF) is one of the most globally prevalent psychostimulants worldwide [9] and is commonly found in coffee, tea, chocolate, energy drinks, and nutritional supplements such as guarana and kola [10]. CAF is rapidly absorbed into the circulatory system, typically reaching peak blood concentrations within one hour (15–120 min) [11]. Since its removal from the World Anti-Doping Agency’s (WADA) prohibited list in 2004 [12], CAF use among athletes has increased markedly, and it continues to be monitored by WADA to track patterns of use and potential misuse [13]. CAF has been shown to improve performance in several sports, across aerobic [14], anaerobic [15,16], and intermittent or mixed-effort sports [17], although responses vary between individuals due to the dosage and the methods employed for measurement [18]. CAF is also suggested to have antioxidant and anti-inflammatory properties, although these effects are less well-known compared with its performance-enhancing actions [19]. Moderate doses (3–6 mg/kg) are sufficient to enhance performance, whereas larger doses (>9 mg/kg) provide limited further benefit and may increase adverse effects [20].

The ergogenic effects of CAF operate via both cerebral and peripheral pathways [16]. Centrally, CAF inhibits adenosine receptors, primarily A1 and A2a, leading to reduced fatigue and sleepiness [21], accelerated neurotransmission, and an increased recruitment of motor units [22]. Peripherally, CAF enhances the mobility of calcium ions and inhibits phosphodiesterase activity, hence improving excitation–contraction coupling in skeletal muscle [16].

Despite the ergogenic effect, CAF’s antagonism of adenosine receptors may negatively affect sleep, especially when consumed later in the day [23,24,25]. Adenosine also plays a key role in sleep regulation, and CAF intake has been associated with delayed sleep onset, reduced total sleep time, and impaired sleep quality [26,27]. Such sleep disturbance may negatively affect recovery and next-day performance [28,29]. In contrast, MEL is a sleep aid that facilitates the onset of sleep and regulates the body’s natural circadian rhythm [30]. These opposite physiological actions prompted us to consider if it is detrimental to combine these two elements or if they function more effectively when administered at separate intervals. Recent research indicates that the combination of certain components may significantly influence their collaborative performance. For example, consuming MEL before sleep may facilitate rest and recovery, while ingestion of CAF before exercise preserves the performance-enhancing effects. In certain instances, delayed dosing may have synergistic effects, enabling athletes to obtain the ergogenic advantages of CAF while maintaining or enhancing their sleep quality with MEL. However, to the best of our knowledge, this potential synergy remains unexamined in the literature; no studies have directly addressed their combined effects on exercise performance, recovery, and sleep, despite the large body of research examining these compounds individually.

The objective of this study was to examine the effects of MEL, CAF, and their combination on sleep, physical, physiological, biochemical, and perceptual results in trained athletes. We hypothesized that the nocturnal MEL ingestion would influence objective sleep parameters during the night of ingestion, and that the combined ingestion of MEL and CAF would more effectively enhance physical and physiological performance, while attenuating fatigue, muscle damage, and inflammation the following morning, compared with either substance alone or placebo.

## 2. Materials and Methods

### 2.1. Participants

**Figure 1:**
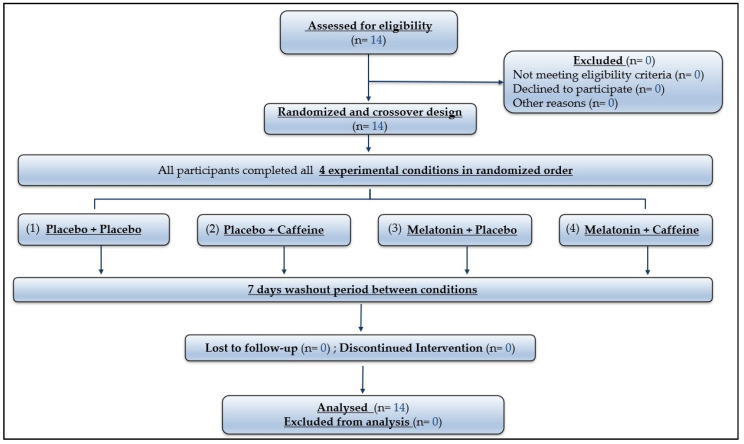
Participants’ flow diagram.

G*Power software (Version 3.1.9.4, University of Kiel, Kiel, Germany) was used to estimate the sample size required for the study for a repeated measures ANOVA test, with four conditions. Previous studies on physical and biochemical outcomes reported an effect size of f = 0.6 with two conditions, α = 0.05, and a statistical power of 80% [31,32]. Given our four-condition design, a slightly smaller effect size (f = 0.5) and higher statistical power (95%) were chosen to ensure robustness of sample size estimation and reduce the risk of type II error. The required sample size was estimated at 10 participants. Considering that some athletes could drop out, 14 trained males (Mean ± SD; age: 22.36 ± 2.9 years; height: 1.81 ± 0.05 m; body mass: 73.5 ± 8.73 kg) were deemed eligible and volunteered to participate in the present study. The participants’ flow diagram is presented in Figure 1. Participants were athletes from team sports or short-term individual sports who routinely performed high-intensity intermittent exercise twice per week and were accustomed to the shuttle run. They were also recruited based on the following inclusion criteria: (a) were non-smokers and non-alcoholics; (b) were moderate caffeine consumers, operationally defined as habitual caffeine intake below 3 mg/kg/day, assessed using a validated caffeine intake questionnaire [33], consistent with caffeine consumption categorization approaches reported in the literature [34]; (c) were classified as trained (3 times/week) [35]; (d) did not suffer from any chronic pathology (e.g., cardiovascular, renal, or liver diseases); (e) did not use dietary supplements or anti-inflammatory drugs; (f) had not taken psychotropic drugs or MEL in the 3 months prior the study; (g) were either ‘moderately morning type’ or ‘neither type’ depending on their answers to the Horne and Ostberg self-assessment questionnaire [36]; (h) had not made trans-meridian journeys in the month prior to the study; and (i) reported no history of sleep disorders or current sleep disturbances. All participants were informed about the procedures, including the possible risks and discomfort involved in this investigation, and they signed a written informed consent form before participating. Our study was conducted according to the last Declaration of Helsinki and fully approved by a local ethical committee (CPP SUD N° 0411/2022) (approval date: 17 May 2022) before the commencement of the experimentation.

### 2.2. Experimental Design

In order to investigate the effects of MEL and CAF supplementation on physical, biochemical, and physiological responses, sleep, and perceived exertion, a randomized double-blind placebo-controlled crossover study design was used following the Consolidated Standards of Reporting Trials (CONSORT) guidelines for a randomized crossover trial [37] (Supplementary Table S1). The order of the experimental conditions was randomized using Research Randomizer (Version 4.0) [38] by an independent researcher not involved in data collection. Before performing the experimental sessions, participants were asked to avoid strenuous exercise, maintain consistent sleep schedules throughout the experimental period, and refrain from caffeine consumption for 2 days prior to testing to reduce residual CAF effects. Sleep–wake patterns were objectively assessed during the night following MEL or PLA ingestion in each experimental session, using actigraphy. Participants were also instructed to maintain consistent dietary intake by completing a dietary record in the 24 h preceding the first trial, which was subsequently replicated before each condition. Additionally, they were familiarized with the testing procedures and had their anthropometric measurements taken 72 h before the commencement of the experimental sessions. No capsules were administered during this baseline assessment.

After the familiarization session, participants performed four different conditions in a randomized order, each separated by a 7-day washout period to minimize potential carryover effects. This duration was chosen to ensure complete elimination of MEL [8] and CAF [39] based on their known pharmacokinetic profiles and relatively short half-lives [40,41]. The four conditions were as follows: (1) PLA + PLA: participants ingested placebo capsules before sleep and placebo capsules in the morning, serving as the full control condition; (2) PLA + CAF: participants ingested placebo before sleep and caffeine (3 mg·kg^−1^) in the morning to isolate the acute effects of caffeine on performance and physiological responses; (3) MEL + PLA: participants ingested melatonin (6 mg) before sleep and placebo in the morning to examine the isolated effects of nocturnal melatonin on sleep and next-day performance; and (4) MEL + CAF: participants ingested melatonin before sleep followed by caffeine in the morning to evaluate the combined effects of melatonin and caffeine. The MEL and CAF doses were determined, respectively, based on previous studies [42,43]. All the sessions were scheduled in the morning hours to avoid any diurnal variations of the performance (from 8:00 a.m. to 10:00 a.m.). To ensure blinding and prevent identification across conditions, capsules were identical in appearance, and CAF and PLA solutions were provided in opaque, unmarked containers. In addition, participants were instructed not to discuss or compare tastes or make assumptions about what they had ingested [39]. Exercise testing was performed one hour after morning ingestion, and an interval was chosen as optimal for full absorption of CAF, allowing its peak concentration to be reached [39,44].

### 2.3. Experimental Protocol

During each testing session, participants were asked to arrive at the sports center no later than 8:30 p.m. and were allowed to prepare themselves to go to bed in a quiet, darkened room. One hour later and 30 min before the scheduled sleep onset at 10.00 p.m., they ingested either 6 mg (2 capsules of 3 mg) of MEL (vegetable source made by Webber Naturals, Coquitlam, BC, Canada) or placebo MEL (PLA; made from lactose, starch, and cellulose). An Actigraph GT3X device was placed on the non-dominant arm (Actigraph, Pensacola, FL, USA) to objectively assess sleep–wake patterns during the night of MEL/PLA ingestion.

The next day, participants were awakened at 7:00 a.m. After consuming standardized breakfast starting at 7:30 a.m., they ingested 3 mg/kg of body mass of caffeine (CAF) at 8:00 a.m., or placebo caffeine (PLA; all-purpose bleached flour) diluted in 200 mL of water. After CAF ingestion, athletes rested quietly for 50 min, after which blood samples were collected to assess biochemical markers. Participants then completed a standardized 5 min warm-up consisting of 2 min of easy running followed by 3 min of dynamic exercises [45], and subsequently performed the 5 m shuttle run test (5mSRT).

**Figure 2:**
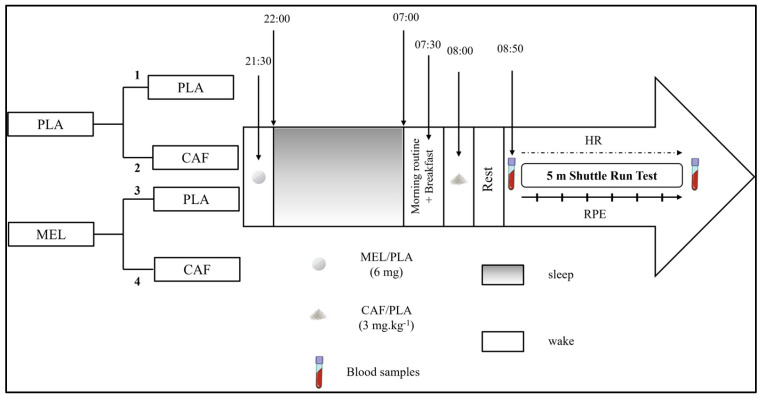
Schematic representation of the study design. MEL: melatonin; PLA: placebo; CAF: caffeine; RPE: rating of perceived exertion; HR: heart rate. 1: PLA + PLA (placebo in both evening and morning); 2: PLA + CAF (placebo in the evening and caffeine in the morning); 3: MEL + PLA (melatonin in the evening and placebo in the morning); 4: MEL + CAF (melatonin in the evening and caffeine in the morning).

Immediately after each repetition of the 5mSRT, the participant rated his perceived exertion (RPE) using the Borg CR-10 scale [46] and then returned to the starting position. Ratings of perceived exertion (RPE) were recorded for each repetition and summed to calculate a mean RPE value for analysis. Heart rate (HR) was monitored continuously throughout the test, and peak heart rate (HR_peak_) was used for analysis. Post-exercise blood samples were collected immediately after completion of 5mSRT to assess markers of muscle damage (creatine kinase (CK), lactate dehydrogenase (LDH), aspartate aminotransferase (ASAT), and alanine aminotransferase (ALAT)) and inflammation (C-reactive protein (CRP)). This protocol is illustrated in Figure 2.

### 2.4. Data Collection and Analysis

#### 2.4.1. Actigraph Registration and Analysis

Participants were asked to wear the GT3X activity monitor on the wrist of the non-dominant arm (ActiGraph, Pensacola, FL, USA), validated by Kushida et al. [47]. Actilife 6 software was used to analyze and calculate the following recorded data: in bedtime (IB), out bedtime (OB), sleep latency (SL), sleep efficiency (SE), total time in bed (TTB), total sleep time (TST), and wake after sleep onset (WASO). The sleep–wake cycle of participants was assessed only during the night of MEL/PLA ingestion.

#### 2.4.2. The 5 M Shuttle Run Test (5mSRT)

The 5mSRT was used to assess high-intensity intermittent performance, including speed and change of direction ability, simulating aerobic and anaerobic pathways [48]. As described previously [48], the protocol consisted of six repetitions of 30 s maximal shuttle sprints, with 35 s of passive recovery period between repetitions. The running distance increased progressively across repetitions as participants repeatedly dashed back and forth over the shuttle course. During the test, the following indices were determined [48]:

(1) Best distance (BD) (m): the greatest distance covered during a 30 s shuttle.
(2) Total distance (TD) (m): the total distance covered during the six 30 s shuttles.
(3) Fatigue index (FI) (%) = [(((Shuttle 1 + Shuttle 2)/2) − ((Shuttle 5 + Shuttle 6)/2)))/((Shuttle 1 + Shuttle 2)/2)] × 100.

#### 2.4.3. Heart Rate (HR)

Heart rate (HR) was recorded every 5 s throughout the 5mSRT (Polar team2 Pro System, Polar Electro OY, Kempele, Finland), and HR_peak_ was determined for subsequent analysis.

#### 2.4.4. Rating of Perceived Exertion (RPE): Assessment Procedure

The subjective RPE score was provided after each repetition of the 5mSRT via an 11-point scale ranging from 0 (very, very easy) to 10 (extremely hard) [46]. The mean score was reported in the statistical analysis and was calculated using the following formula [45]:

RPE (a.u.) = Σ (RPE scores)/number of repetitions

#### 2.4.5. Blood Sample and Variables Analysis

Blood sampling was collected from a forearm vein after 5 min of resting before the 5mSRT and again 5 min following the test. Two types of tubes were used: heparinized tubes (Becton Dickinson, Franklin Lakes, NJ, USA) for plasma separation to determine CK, LDH, ASAT, and ALAT; and tubes without anticoagulant (Becton Dickinson, Franklin Lakes, NJ, USA) for serum collection to determine CRP. Plasma was isolated by centrifugation at 4000 rpm within 15 min of sample collection. All biochemical tests were performed on an automated analyzer (Selectra Pro XL, ELITechGroup, Puteaux, France) in accordance with standard laboratory protocols. CK, LDH, ASAT, and ALAT were quantified using commercial reagent kits (ELITechGroup, Puteaux, France) by spectrophotometric or kinetic methods at 340 nm, while CRP was assessed using the immunoturbidimetric assay (ELITechGroup, Puteaux, France). The intra-assay coefficient of variation (CV) for the measured parameters was 1.3%, 0.2%, 1.1%, 1.5%, and 1.16%, respectively.

### 2.5. Statistical Analysis

The statistical analysis was performed using the SPSS 27.0 statistical software (IBM corps, Armonk, NY, USA). Data are presented as the mean and standard deviation for variables with normal distribution and median and interquartile range for non-normally distributed variables. The Shapiro–Wilk test was used to check and confirm the normality of data sets, and the Levene test was used to verify the homogeneity of variances. Sphericity was tested using the Mauchly test. For variables with normal distribution, a two-way (condition × time) or one-way (condition) analysis of variance (ANOVA) with repeated measurements was used, with Bonferroni correction being used as the post hoc test. Standardized effect size (Cohen’s d) was used to interpret the magnitude of differences between variables and considered as: trivial (≤0.20); small (≤0.60); moderate (≤1.20); large (≤2.0); very large (≤4.0); and extremely large (>4.0) [49]. For the variables with non-normal distribution, the non-parametric Friedman test was used, with the Wilcoxon signed rank test used as a post hoc test. The rank biserial correlation coefficient (r) was calculated and considered as 0.1 to <0.3 (small), 0.3 to <0.5 (moderate), and ≥0.5 (large) [50,51]. The level of statistical significance was set at *p* ≤ 0.050.

## 3. Results

### 3.1. Sleep Parameters

Regarding sleep parameters, no significant effect of conditions was observed for any parameters (all *p* > 0.05). A one-way repeated-measures ANOVA revealed no significant differences across conditions for: in bedtime (F_3,39_ = 0.558; *p* = 0.646; η_p_^2^ = 0.41), sleep efficiency (F_1.71,22.22_ = 2.649; *p* = 0.100; η_p_^2^ = 0.169), total time in bed (F_3,39_ = 1.074; *p* = 0.371; η_p_^2^ = 0.76), and total sleep time (F_1.69,21.98_ = 1.355; *p* = 0.275; η_p_^2^ = 0.94). Similarly, Friedman analyses showed no significant condition effects for: out bedtime (χ^2^_(3)_ = 1.187; *p* = 0.756), sleep latency (χ^2^_(3)_ = 7.413; *p* = 0.060), and wake after sleep onset (χ^2^_(3)_ = 2.000; *p* = 0.572) (Table 1).

### 3.2. Physical Performance During the 5mSRT, Related HR_peak_, and RPE Values

#### 3.2.1. Total Distance

Pairwise comparisons revealed that PLA + PLA elicited lower values than PLA + CAF (z = −2.86; r = 0.76; *p* = 0.004), MEL + PLA (z = −3.11; r = 0.83; *p* = 0.002), and MEL + CAF (z = −3.18; r = 0.85; *p* = 0.001) (Table 2).

#### 3.2.2. Best Distance

Pairwise comparisons showed that PLA + PLA and MEL + PLA induced lower values than MEL + CAF (z = −2.94; r = 0.76; *p* = 0.003 and z = −2.70; r = 0.72; *p* = 0.007). Moreover, PLA + PLA resulted in lower values than PLA + CAF (z = −2.09; r = 0.56; *p* = 0.036) (Table 2).

#### 3.2.3. Fatigue Index

Higher values were recorded under MEL + CAF than MEL + PLA (95%CI_diff_ = 4.33 to 19.20; *p* = 0.002; d = 1.87) and PLA + CAF (95%CI_diff_ = 2.12 to 15.24; *p* = 0.007; d = 1.27). Moreover, PLA + PLA elicited higher values than MEL + PLA (95%CI_diff_ = 5.85 to 20.30; *p* < 0.001; d = 1.95) and PLA + CAF (95%CI_diff_ = 1.01 to 18.18; *p* = 0.025; d = 1.37) (Table 2).

#### 3.2.4. Peak Heart Rate

HR_peak_ differed significantly across conditions (χ^2^_(3)_ = 7.92; *n* = 14; *p* = 0.048). Pairwise comparisons revealed that PLA + PLA elicited higher values than MEL + CAF (z = −2.67; r = 0.71; *p* = 0.008) and MEL + PLA (z = −1.99; r = 0.53; *p* = 0.047). Moreover, PLA + CAF elicited higher values than MEL + CAF (z = −2.04; r = 0.55; *p* = 0.041) (Table 2).

#### 3.2.5. Rating of Perceived Exertion

A significant main effect of condition was observed for RPE (F_3,39_ = 3.14; *p* = 0.036; η_p_^2^ = 0.19). However, pairwise comparisons did not reveal significant differences between conditions (Table 2).

### 3.3. Biochemical Parameters

#### 3.3.1. Aspartate Aminotransferase

**Figure 3:**
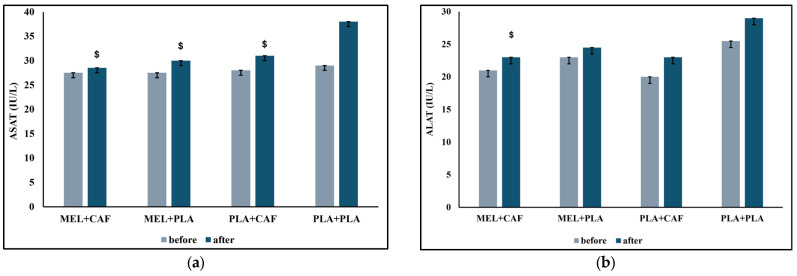

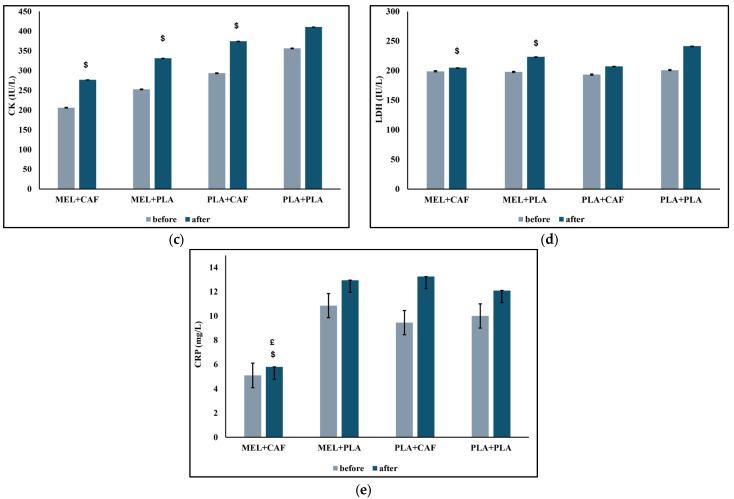
Biochemical parameters recorded before and 5 min after the 5 m shuttle run test (5mSRT) across four conditions combining nocturnal melatonin (MEL) or placebo ingestion before sleep and morning caffeine (CAF) or placebo ingestion before the onset of the test (*n* = 14). (**a**) ASAT values recorded before and after 5 min after the 5mSRT across the four conditions; (**b**) ALAT values recorded before and after 5 min after the 5mSRT across the four conditions; (**c**) CK values recorded before and after 5 min after the 5mSRT across the four conditions; (**d**) LDH values recorded before and after 5 min after the 5mSRT across the four conditions; (**e**) CRP values recorded before and after 5 min after the 5mSRT across the four conditions. Differences between conditions only are indicated: ^$^ significant difference compared with post PLA + PLA (*p* < 0.05). ^£^ significant difference compared with post MEL + PLA (*p* < 0.05). PLA: placebo; MEL: melatonin; CAF: caffeine; CK: creatine kinase; LDH: lactate dehydrogenase; ASAT: aspartate aminotransferase; ALAT: alanine aminotransferase; CRP: C-reactive protein.

The Friedman test indicated a significant effect of time on ASAT values across conditions (χ^2^_(7)_ = 37.06; *p* < 0.001). The Wilcoxon test showed that pre-MEL + CAF values were lower than those post-MEL + CAF (z = −2.51; r = 0.67; *p* = 0.012). In addition, pre-PLA + CAF values were lower than those post-PLA + CAF (z = −3.33; r = 0.89; *p* = 0.001). Furthermore, pre-MEL + PLA values were lower than those post-MEL + PLA (z = −3.08; r = 0.82; *p* = 0.002). Also, pre-PLA + PLA values were lower than post-PLA + PLA (z = −3.24; r = 0.87; *p* = 0.001). There is no significant difference between conditions before exercise. However, comparisons among conditions post-exercise revealed that ASAT levels were lower in MEL + CAF (z = −2.95; r = 0.79; *p* = 0.003), MEL + PLA (z = −2.11; r = 0.56; *p* = 0.035), PLA + CAF (z = −2.42; r = 065; *p* = 0.016) compared with PLA + PLA (Figure 3).

Analysis of pre-to-post exercise changes (∆ = post − pre) confirmed these findings, showing a significantly lower increase in ASAT in the MEL + CAF condition compared with PLA + CAF (*p* = 0.011) and PLA + PLA (*p* = 0.006). In addition, MEL + PLA and PLA + CAF conditions also showed lower increases compared with PLA + PLA (*p* = 0.003; *p* = 0.019, respectively).

#### 3.3.2. Alanine Aminotransferase

There was a significant difference among the distributions of time (χ^2^_(7)_ = 28.15; *n* = 14; *p* < 0.001). Pairwise comparisons revealed that pre-MEL + CAF elicited lower values than post-MEL + CAF (z = −3.37; r = 0.90; *p* < 0.001). In addition, pre-MEL + PLA values were lower than those post-MEL + PLA (z = −3.32; r = 0.89; *p* < 0.001). Moreover, pre-PLA + CAF values were lower than those post-PLA + CAF (z = −3.21; r = 0.86; *p* = 0.001). A similar difference were observed between pre-PLA + PLA values and post-PLA + PLA (z = −3.20; r = 0.86; *p* = 0.001). There is no significant difference between conditions before exercise. However, ALAT levels were lower in post-MEL + CAF than those of post-PLA + PLA (z = −2.04; r = 0.55; *p* = 0.041) (Figure 3).

Analysis of pre-to-post exercise changes (∆ = post − pre) further extended these findings, showing a significantly lower increase in ALAT in the MEL + CAF condition compared with PLA + PLA, MEL + PLA and PLA + CAF (*p* = 0.006; *p* = 0.016; *p* = 0.046, respectively).

#### 3.3.3. Creatine Kinase

The two-way ANOVA test showed a significant effect of time (F_1,13_ = 12.84; *p* = 0.003; ηp^2^ = 0.50), with higher values recorded at post-exercise compared with pre-exercise (95%CI_diff_ = 43.49 to 175.61; *p* = 0.003; d = 2.08). Moreover, there was a significant effect of condition (F_3,39_ = 24.72; *p* < 0.001; ηp^2^ = 0.66) with no significant interaction between time and condition (F_3,39_ = 1.59; *p* = 0.23; ηp^2^ = 0.11). Pairwise comparison showed that the MEL + CAF condition elicited lower values than MEL + PLA (*p* < 0.001), PLA + CAF (*p* = 0.001) and PLA + PLA (*p* < 0.001). Furthermore, MEL + PLA and PLA + CAF elicited lower values than PLA + PLA (*p* = 0.025 and *p* < 0.001, respectively) (Figure 3).

Analysis of pre-to-post exercise changes (∆ = post − pre) partially confirmed the main finding, showing a significantly lower increase in CK in the MEL + CAF condition compared with MEL + PLA, PLA + CAF, and PLA + PLA (*p* = 0.011; *p* = 0.011; *p* = 0.019, respectively).

#### 3.3.4. Lactate Dehydrogenase

LDH differed significantly across conditions (χ^2^_(7)_ = 37.81; *n* = 14; *p* < 0.001). Pairwise comparisons revealed that LDH values were lower at pre- compared with post-test for MEL + CAF (z = −3.31; r = 0.88; *p* < 0.001), PLA + CAF (z = −3.30; r = 0.88; *p* < 0.001), and MEL + PLA (z = −3.18; r = 0.85; *p* = 0.001). Moreover, lower values were recorded at post-test for MEL + CAF and MEL + PLA conditions and at pre-test for the PLA + PLA condition compared with values at post-test for the PLA + PLA condition (z = −2.48; r = 0.66; *p* = 0.013; z = −2.64; r = 0.71; *p* = 0.008 and z = −3.30; r = 0.88; *p* < 0.001, respectively) (Figure 3).

Analysis of pre-to-post exercise changes (∆ = post − pre) confirmed these findings, showing a significantly lower increase in LDH in the MEL + CAF, MEL + PLA, and PLA + CAF conditions compared with PLA + PLA (*p* = 0.007; *p* = 0.003; *p* = 0.003, respectively).

#### 3.3.5. C-Reactive Protein

CRP differed significantly across conditions (χ^2^_(7)_ = 25.15; *n* = 14; *p* < 0.001). Pairwise comparisons revealed that values at pre- were lower than those at post-test for the MEL + CAF (z = −2.04; r = 0.55; *p* = 0.041), MEL + PLA (z = −2.24; r = 0.60; *p* = 0.025), and PLA + PLA (z = −3.17; r = 0.85; *p* = 0.002) conditions. Moreover, there were lower values at post-test for the MEL + CAF condition compared with MEL + PLA (z = −2.00; r = 0.53; *p* = 0.045) and PLA + PLA (z = −2.34; r = 0.63; *p* = 0.019) (Figure 3).

Analysis of pre-to-post exercise changes (∆= post − pre) further supported these findings, showing a significantly lower increase in CRP in the MEL + CAF condition compared with MEL + PLA, PLA + CAF, and PLA + PLA (*p* = 0.041; *p* = 0.019; *p* = 0.03, respectively).

## 4. Discussion

This study examined the potential synergistic effects of nocturnal melatonin ingestion (6 mg administered before sleep) and morning caffeine ingestion (3 mg·kg^−1^ administered 1 h before exercise) on sleep quality and quantity, biochemical markers, physical performance, physiological responses, and perceived exertion in trained males. Regarding sleep, no variations were observed between conditions. Concerning exercise performance, all active conditions (PLA + CAF, MEL + PLA, and MEL + CAF) increased TD during the 5mSRT compared with the full PLA condition (PLA + PLA). CAF-containing trials (PLA + CAF and MEL + CAF) were associated with greater BD, whereas reductions in FI were observed only with MEL and CAF administered separately. In addition, MEL ingestion before sleep (MEL + PLA and MEL + CAF) was associated with lower peak HR during exercise. Finally, the combined MEL + CAF condition was associated with low markers of muscle damage and inflammation compared with either supplement alone or PLA. A significant main effect of condition on perceived exertion was observed, although no pairwise differences reached significance. From a practical perspective, these findings indicate that nocturnal melatonin did not significantly improve sleep quality or quantity; it may support next-day high-intensity performance and reduce physiological strain, and combining melatonin with caffeine may help preserve performance related-outcomes.

The present study showed no significant effect of nocturnal MEL ingestion on objective sleep parameters. This is consistent with previous investigations reporting no changes in sleep variables following the ingestion of 5 mg and 6 mg of MEL administered 30 min before bedtime [32,52]. In contrast, other studies have shown improvements in subjective sleep quality following higher MEL doses (8–10 mg) [7,53]. Differences between studies may be related to several factors. Recent meta-analyses [54] indicate that the effects of MEL on sleep are generally modest, depending on the study design and population’s characteristics. Improvements are more consistently observed for sleep onset latency and total sleep time, whereas other parameters, such as sleep efficiency and sleep quality, show more variable responses. From a mechanistic perspective, MEL primarily functions as a circadian regulator rather than a direct hypnotic agent, potentially limiting its impact on objective sleep measures, especially in individuals without sleep disturbances. Accordingly, the trained status and likely optimal baseline sleep quality of the participants in the present study, who are characterized by regular sleep–wake schedules and healthy circadian rhythms, may have resulted in a ceiling effect, reducing the potential for measurable improvements. Furthermore, the timing of ingestion relative to endogenous MEL secretion may affect its effectiveness, as taking it close to habitual bedtime may provide limited additional benefit when endogenous levels are already elevated. All these factors may partly explain the absence of significant effects observed in the present study.

Considering physiological and physical performance, this study emphasizes decreased HR_peak_ and enhanced 5mSRT performance (i.e., TD, BD) with MEL, CAF, or their combination in comparison with PLA. A more significant decrease in FI was observed only in the separate MEL and CAF conditions. To the best of our knowledge, no prior research has investigated the synergistic effects of MEL and CAF, and previous results for each supplement individually are inconsistent. Variations in MEL’s effects may arise from methodological differences, such as dosage, timing, exercise protocols, and participants’ fitness levels [53]. Certain studies indicated that nocturnal MEL enhanced both short-term and endurance performance [7,8,53,55], while other research noticed no performance advantages following nocturnal [52,56,57] or diurnal [3,58,59,60,61] MEL administration. The impact of CAF on physical performance is well researched yet inconsistent. Certain studies indicated performance enhancements, especially with dosages of 3–6 mg/kg in endurance activities [62,63,64], muscular strength [15,65,66], and sport-specific intermittent high-intensity efforts [17]. Conversely, several studies have not identified substantial enhancements [67,68], potentially attributable to untrained individuals lacking familiarity with intermittent-sprint exercise [19]. Concerning HR, only the assessment of Atkinson et al. [58] observed a minor reduction of 6–9 beats·min^−1^ following daytime administration of 5 mg MEL, while other investigations reported no significant difference between MEL and PLA [7,58,59,69,70,71]. The reduction in heart rate after MEL consumption has been associated with diminished sympathetic activity [72] and decreased catecholamine levels [73]. A significant main effect of conditions on RPE was observed during the 5mSRT; however, post hoc comparisons did not reveal a significant pairwise difference. This discrepancy reflects limited statistical power due to the modest sample size. Previous studies generally report no substantial effect of MEL on RPE [52,56,57,58,59,74]. However, dose-dependent effects have been suggested, with Ghattassi et al. [53] reporting decreased RPE following 8 mg of MEL, while Souissi et al. [71] recorded increased RPE at 6 mg. Similarly, the effects of CAF on RPE are inconsistent, with meta-analyses indicating no significant influence during resistance exercise [75] but potential reductions during submaximal and aerobic exercise [76,77]. The high-intensity nature of 5mSRT may have attenuated perceptual sensitivity to supplementation, potentially masking condition-specific differences. Furthermore, the CAF dosage employed may have been insufficient to influence perceptual responses under these demanding conditions.

As anticipated, concentrations of muscle damage indicators (ASAT, ALAT, CK, LDH) and inflammation (CRP) increased after the 5mSRT, consistent with prior studies documenting comparable responses after high-intensity exercise [78,79,80,81]. The rapid post-test increase supports the existence of exercise-induced muscle damage [82]. This study provides preliminary evidence that multiple biochemical indicators of muscle damage and inflammation were lower following the 5mSRT in the MEL + CAF condition compared with PLA + PLA and the individual supplements. MEL + CAF significantly decreased ASAT and ALAT levels, corroborating the hypothesis that the synergistic effects of MEL and CAF can mitigate muscle damage following intense exercise. The isolated administration of MEL and CAF also diminished ASAT levels compared with PLA, indicating a possible degree of protective effect from each supplement independently. Furthermore, LDH and CRP levels decreased not only in the MEL + CAF group but also in the MEL + PLA and CAF conditions, indicating that MEL independently provides muscle-protective and anti-inflammatory benefits. Nonetheless, the combination yielded greater reductions, especially for CK, than either supplement individually. These findings were further supported by the analysis of pre-to-post exercise changes, which generally confirmed a greater reduction in muscle damage and inflammatory responses in the MEL + CAF condition. Direct comparisons with previous studies are challenging due to the absence of research on the acute effects of MEL + CAF co-ingestion. Our findings, when assessing each supplement individually, align with studies indicating that MEL taken prior to bedtime or exercise [8,56,69] reduces increases in cellular damage and/or inflammation. Conversely, research on CAF and exercise-induced muscle damage is scarce [83] and presents inconclusive findings: some studies indicate that CAF may exacerbate muscle damage [84,85], while others demonstrated no significant impact of CAF alone on muscle damage markers [86,87,88,89,90]. Despite being statistically significant with moderate-to-large effect sizes, these changes likely reflect normal physiological responses to high-intensity exercise rather than meaningful reductions in muscle damage and should therefore be interpreted with caution. In addition, ASAT and ALAT are not exclusively specific to skeletal muscle, which may limit their interpretation as direct muscle damage. Future research is needed to determine whether these effects lead to improved recovery or reduced delayed onset muscle soreness (DOMS) in order to better clarify the true practical utility of MEL + CAF ingestion.

Overall, the performance responses observed across conditions suggest that both nocturnal melatonin and morning caffeine may independently influence next-day exercise capacity through complementary and potentially synergetic mechanisms. The increase in total distance observed in PLA + CAF is likely explained by the well-established stimulatory effects of caffeine on the central nervous system, including enhanced motor unit recruitment, increased alertness, and reduced perception of effort, which are known to facilitate high-intensity intermittent performance. In contrast, the performance benefits observed in MEL + PLA may be partly related to melatonin regulatory effects on autonomic balance, as reflected by the lower peak heart rate responses reported in Table 2. Importantly, the greater magnitude of some performance outcomes observed in the combined condition (MEL + CAF) likely reflects an additive or synergistic interaction between improved nocturnal recovery and enhanced morning arousal. This interpretation is further supported by the biochemical responses reported in Figure 3, where MEL + CAF was associated with more favorable profiles of markers of muscle damage and inflammation, suggesting a potential attenuation of physiological strain for a given performance output. Collectively, these findings indicate that optimizing both recovery-related processes during sleep and arousal-related mechanisms before exercise may provide a more effective strategy to enhance performance than targeting either pathway in isolation, although further research is required to confirm these effects.

Several limitations warrant consideration. Initially, the relatively small sample size may limit the generalizability of the findings and, together with the number of outcomes assessed, requires cautious interpretation of the results. Additionally, we assessed the acute effects of MEL + CAF solely, leaving the long-term implications of this combination uncertain. Secondly, we administered a singular dosage of each supplement; varying dosages or timing of use may produce different results. Third, we did not evaluate delayed biochemical reactions (e.g., up to 72 h), which could offer additional information on their interaction beyond the immediate post-administration phase. Baseline differences in biochemical markers across conditions may have influenced the post-exercise values, so the interpretation of change-score (∆) results should be considered with caution. In addition, sleep was monitored across conditions; however, only the night of the MEL/PLA ingestion was analyzed, which may reduce the sensitivity of the assessment and the interpretation of sleep-related responses. Consequently, the specific effect of morning CAF intake on subsequent sleep cannot be independently isolated in the present design. Finally, endogenous MEL levels were not assessed. Therefore, it is not possible to determine whether individual differences in baseline MEL influenced the observed responses to supplementation. Additionally, other pertinent factors, including body temperature and cortisol, were not evaluated.

Future research should investigate the long-term effects of MEL + CAF, examine various dosages to elucidate potential dose–response relationships and enhance utilization, and involve larger, more diverse samples encompassing both sexes. As participants in the present study did not indicate sleep disorders, subsequent research should examine this relationship in individuals with sleep disturbances who may experience more significant benefits. Furthermore, future studies should consider assessing sleep across several nights and/or incorporating subjective sleep assessments to offer a more comprehensive evaluation of sleep responses. In particular, the impact of CAF alone or in combination with MEL on sleep should be specifically investigated using appropriately timed sleep assessment. Finally, it would be useful for future studies to implement a longer or more standardized caffeine washout period before testing to better control for residual effects. In addition, they may compare responses under fasted and fed conditions to understand the impact of nutritional status.

## 5. Conclusions

The nocturnal administration of 6 mg of melatonin followed by the morning ingestion of 3 mg·kg^−1^ of caffeine was associated with greater improvements than either supplement alone or placebo, notably enhancing certain aspects of physical and physiological performance during high-intensity, repetitive exercise, accompanied by favorable changes in selected markers of muscle damage and inflammation. Given that these effects were not consistent across all performance outcomes, and no influence on sleep was observed, these results should be interpreted cautiously. MEL + CAF seems to be a promising approach to improve athletic performance, though its effects on sleep necessitate additional research.

## Figures and Tables

**Table 1:** Sleep parameters recorded in four conditions combining nocturnal melatonin (MEL) or placebo ingestion before sleep and morning caffeine (CAF) or placebo ingestion before the onset of the test (*n* = 14).

	MEL + CAF	MEL + PLA	PLA + CAF	PLA + PLA	Statistics
In bedtime (hh:min)	22:17 ± 0:23	22:29 ± 0:14	22:26 ± 0:42	22:30 ± 0:49	F_3,39_ = 0.558; *p* = 0.646; η_p_^2^ = 0.41
Out bedtime (hh:min)	07:00 (0:22)	06:55 (0:08)	07:04 (0:35)	06:56 (0:08)	χ^2^_(3)_ = 1.187; *p* = 0.756
Sleep latency (min)	31 (119)	58.5 (82)	64 (103)	61 (69)	χ^2^_(3)_ = 7.413; *p*= 0.060
Sleep efficiency (%)	61.38 ± 9.82	65.10 ± 4.77	56.59 ± 9.87	56.07 ± 19.04	F_1.71,22.22_ = 2.649; *p* = 0.100; η_p_^2^ = 0.169
Total time in bed (min)	528.21 ± 50.36	505.93 ± 32.68	494.21 ± 56.80	506.36 ± 58.71	F_3,39_ = 1.074; *p* = 0.371; η_p_^2^ = 0.76
Total sleep time (min)	320.93 ± 67.81	322.36 ± 40.27	278.86 ± 54.52	317.14 ± 89.26	F_1.69,21.98_ = 1.355; *p* = 0.275; η_p_^2^ = 0.94
Wake after sleep onset (min)	145 (110.5)	121 (89)	118.5 (124.75)	124 (105.5)	χ^2^_(3)_ = 2.000; *p* = 0.572

PLA: Placebo; MEL: melatonin; CAF: caffeine; hh: 2-digit hours; min: minutes. Values are presented as the median (interquartile range) for out bedtime, sleep latency, and wake-up after sleep onset and as the mean ± SD for the other variables.

**Table 2:** Physical performance parameters, peak heart rate (HR_peak_), and rating of perceived exertion (RPE) during the 5 m shuttle run test (5mSRT) recorded in four conditions combining nocturnal melatonin (MEL) or placebo ingestion before sleep and morning caffeine (CAF) or placebo ingestion before the onset of the test (*n* = 14).

	MEL + CAF	MEL + PLA	PLA + CAF	PLA + PLA	Statistics
TD (m)	802.50 (121.50) *	740.00 (56.25) *	752.50 (77.50) *	620.00 (75.00)	χ^2^_(3)_ = 24.11; *p* < 0.001
BD (m)	155.00 (37.50) *^,µ^	130.0 (17.50) *	137.50 (25.00) *	125.00 (18.75)	χ^2^_(3)_ = 19.82; *p* < 0.001
FI (%)	14.65 (15.56) ^µ,£^	3.66 (4.57)	6.33 (7.22)	19.60 (18.45) ^µ,£^	F_1.88,24.42_ = 13.29; *p* < 0.001; η_p_^2^ = 0.51
HR_peak_ (beats·min^−1^)	182 (16) *^,£^	184 (9) *	186 (4)	188 (9)	χ^2^_(3)_ = 7.92; *p* = 0.048
RPE (a.u.)	7.19 ± 1.46	6.37 ± 1.27	5.75 ± 1.77	6.77 ± 1.13	F_3,39_ = 3.14; *p* = 0.036; η_p_^2^ = 0.19

*: Different from PLA + PLA at *p* ˂ 0.05; ^µ^: different from MEL + PLA_CAF_ at *p* ˂ 0.05; ^£^ different from PLA + CAF at *p* ˂ 0.05; PLA: placebo; MEL: melatonin; CAF: caffeine; TD: total distance; BD: best distance; FI: Fatigue index; HR_peak_: peak heart rate; RPE: rating of perceived exertion; a.u.: arbitrary units. Values are presented as the mean ± SD for RPE and as the median (interquartile range) for the other variables.

## Data Availability

The data presented in this study are available on request from the corresponding author due to privacy restrictions, the data are publicly unavailable since they are part of an ongoing project.

## Supplementary Materials

The following supporting information can be downloaded at: https://www.mdpi.com/article/10.3390/nu18091425/s1, Table S1: CONSORT checklist of the study.

## Abbreviations

The following abbreviations are used in this manuscript:

ALAT	Alanine aminotransferase
ASAT	Aspartate aminotransferase
a.u.	Arbitrary units
CAF	Caffeine
CK	Creatine kinase
CRP	C-reactive protein
FI	Fatigue index
HR_peak_	Peak heart rate
LDH	Lactate dehydrogenase
MEL	Melatonin
PLA	Placebo
RPE	Rating of perceived exertion
WASO	Wake after sleep onset
5mSRT	The 5 m shuttle run test
